# Drug Eruptions: Their Nature and Varieties

**Published:** 1911-01-07

**Authors:** J. H. Stowers

**Affiliations:** Vice-President of the Section of Dermatology, Royal Society of Medicine; late Physician to the Department for Diseases of the Skin, N.W. London Hospital; formerly President of the Dermatological Society of Great Britain and Ireland.


### Hospital Clinics.

### DRUG ERUPTIONS: THEIR NATURE AND VARIETIES.

By J. H. STOWERS, M.D., Vice-President of the Section of Dermatology, Royal Society of Medicine; late Physician to the Department for Diseases of the Skin, N.W. London Hospital; formerly President of the Dermatological Society of Great Britain and Ireland.

(Lecture at the Polyclinic, Chenies Street, W.C.)

Gentlemen,—Some years ago, when I was engaged in demonstrating cutaneous disease at St. Bartholomew's Hospital, I found it to be frequently the case that students failed in their diagnosis because they lacked some formulated plan upon which to deal with the patients presented to them. The idea prevailed to a larger extent than was desirable that it was necessary, in order to arrive at a conclusion, that a similar example of disease should have been previously demonstrated. The consequence was that very many arrived at a diagnosis, not always correct, of course, by guess-work alone, for, as you are well aware, a beginner in the department has practically no knowledge of the disorders and diseases with which he is called upon to deal.

### Methods of Physical Examination.

In the study of clinical medicine the first duty of the teacher is to acquaint his pupil with the methods of physical examination, such as inspection, palpation, percussion and auscultation—in other words, to instruct him in the accepted method of investigation before he attempts to undertake the duties of clinical clerk in the out-patient room or the wards.

Careful reading of dermatological subjects in a selected text-book and the study of illustrations in the form of plates or coloured drawings are always useful even to those who have not yet attended the department; but if a diagnosis is to be founded upon reliable and scientific grounds it must be *built up*, as it were, by the recognition of certain characteristic objective signs, or lesions, supported by the subjective symptoms which occur coincidently with them.

In order that the student might have some assistance in this direction I drew up the "Method of Investigation," a copy of which I hand round to you.* As you will see, it includes the salient points necessary for practical investigation and case-taking, for although several modern methods now in vogue were not then in daily use, my sheet requires but little addition to bring it up to date.

### Practical Evidence.

An evidence of the value of the scheme soon occurred in the following incident: A member of my class who was a candidate for his London University M.B. degree, although he failed to diagnose completely one or two of the skin diseases presented to him at the examination, was awarded sufficient marks to secure a pass because of the method and course he adopted in investigating the cases, which favourably impressed the examiners.

When a student commits himself to a diagnosis it is desirable to inquire of him upon what he bases his conclusion. If he is not merely hazarding a guess, but evinces a knowledge of detail which shows that he has recognised the initial lesions in their development and the characteristics of the disease, there can be no doubt of the soundness of his views and the quality of his studies.

### Exactitude in Medicine.

You will forgive me, gentlemen, I am sure, for these preliminary remarks, for, however extensive your dermatological experience may be in general, it is always possible in an audience such as this that there is someone to whom the subject is less familiar, and to him, at any rate, a service may be rendered by describing a method which to my knowledge has been of much practical assistance to practitioners and students for many years. Its adoption has contributed to accuracy and uniformity in note-taking, and besides, when the details are filled in, it affords a record of facts of reliable and instructive character. Everything that tends to exactitude in the practice of medicine is worth consideration, for we shall be agreed that a medical man is often put to a severe test when he has a skin disease of importance to treat, for not only is the patient (and his friends also in many instances) alarmed by the nature and extent of the ailment, but their critical eyes enable them to follow the progress of the case and, in addition, the degree to which it yields to the treatment described and corresponds with the prognosis given.

A patient, as you well know, with acute general eczema, or an extensive exfoliative dermatitis (not such a so-called "rare disease" as was reported in the *Times* last month, which is alleged to have required six doctors and 320 days' detention in a local isolation hospital before the medical officer of the district was informed of the nature of the case), I say a patient with acute general eczema or an extensive exfoliative dermatitis does not depend so much upon the statement of his doctor concerning his improvement as he who is the subject of a functional abdominal disorder, hepatic, renal, or the like, for it is not a rare experience—and we do well to be reminded of it—to hear a patient's feelings expressed concerning his ailment and his adviser in such a manner as to be interpreted thus:—

"If he cureth *not* those disorders which he *doth* see, how much less likely is he to treat successfully those diseases which he doth *not* see?"

Now, gentlemen, if for the moment you will adopt the method of investigation I have advocated we will stay to consider the all-important line of "Previous treatment (effects of remedies), (*a*) General (drug eruptions?). Dermatitis medicamentosa; (*b*) Local (effects of irritants?). Dermatitis venenata." In other words, we will briefly

* See page 436.

study the special topic upon which I desire to found my remarks to-day.

My Choice of a Subject.

When I was invited to address you I selected the subject of "Drug Eruptions: Their Nature and Varieties," as one that could not fail to interest, for wherever the healing art is exercised attention must constantly be devoted to disorders and diseases of the skin, directly or indirectly produced.

Those who are occupied in the practice of general medicine or surgery have no inconsiderable opportunity of witnessing the effects of the innumerable therapeutic agents in daily use. I am not unmindful of the fact that to treat of the nature and varieties of drug eruptions with anything like completeness in the short time allotted to me is impossible, besides which an individual experience of the subject must necessarily be far too limited to allow the inclusion from personal knowledge of all the many eruptions which may be produced by medicaments.

I can only attempt a very brief *résumé* drawn from the writings and observations of others, to which I will add my own experience and the narration of a few of the cases of special interest which have been under my own care.

A Modern Study.

The scientific study of drug eruptions is of comparatively recent date. Our text-books on materia medica and therapeutics, with a few exceptions, make but slight reference to them, and the majority of these include but a few of the best known or commonest varieties. Morrow, of New York, in his excellent book—the most complete, I believe, which has yet been published on the subject—states that Lorry, of Paris, "was the first dermatologist who called attention to the production of eruptions from the use of drugs in a treatise published in 1777." Subsequently Bell, of London, in 1796; Alley, of Dublin, in 1804; Montègre, in 1814; Razer, in 1835; Ricord, in 1842; Devergie, in 1857; and Bazin, in 1862, have followed in the same direction, and more recently many observers in England, America, France, Germany, Austria, and other countries have studied and written upon this important subject.

As would be expected, there is no single definition of drug eruptions which has been universally accepted; indeed, the term is occasionally used in a somewhat different sense by various writers. By some the influences of direct or external contact are omitted, and only those included in the list which follow internal administration. By others all evident and manifest changes in the skin, whether produced by actual and external contact or by internal administration (*i.e.* by the mouth, the rectum, the genito-urinary system, inhalation, or subcutaneous injection—in fact, every channel connected with absorption and elimination), are included in the group. By others again, as Morrow states, the term "has been limited in its application to the anomalous or incidental effects of drugs upon the cutaneous surface in contradistinction to changes in the skin which are a more or less constant expression of the drug's physiological action, and which may be provoked for therapeutical purposes." The proper signification of the term embraces all congestive and inflammatory changes in the skin caused by the external or internal use of drugs.

The Definition of Eruptions.

In this definition every eruption due to outward application must be included, whether for medicinal, therapeutic, or commercial purposes—*i.e.* connected with the various industries in which chemical and other agents are so largely employed, besides all those which result from the introduction of every kind of drug into the system by absorption through whatever structure or channel it may find entrance. If our definition is to be really inclusive and complete we cannot omit the series of cutaneous manifestations which have been observed in connection with inoculation with attenuated virus, either of animal or specific origin, utilised for the conferring of immunity from, or for neutralising the effects of, poisons or toxic products already introduced into the blood. And yet more recently we have recorded, though not strictly, perhaps, within the boundary, the effects attributable to the use of electricity for diagnostic or other remedial purposes, which alter the nutrition and materially disturb the structure of the skin and appendages consequent upon the use of the Röntgen method, including the so-called $x$-ray burn.

Particular Causes.

Electrolysis, too, is sometimes responsible for consequences with which we have to deal. Self-inflicted injuries for the purpose of deception deserve a place in the group, but this section would have to include all those various manifestations resulting from the dealings of persons whose psychological peculiarities require special consideration. Chloroform and ether inhalations are capable of producing remarkable changes of an eruptive character, of which I witnessed examples when anæsthetist at St. Bartholomew's Hospital.

All these are to be included in the ever-increasing list of therapeutic agents which exercise peculiar and obvious effects upon the cutaneous system. The results of parasites, acarus, scabiei, pediculi and others, both animal and vegetable, must be remembered.

Concerning *classification*, Bazin merely divided the group into two, namely: (1) Those produced by direct application upon the integument; (2) those resulting from indirect administration or absorption.

Behrend went further, and classed drug eruptions as follows, namely: (1) Those occasioned by the specific action of a drug—acute in character; (2) those caused by the elimination through the skin of certain drugs—chronic in character; (3) eruptions caused by the dynamic action of drugs which are, like the first class, acute in their development and course, but appear to possess a certain period of incubation.

Notes on their Ætiology.

Dr. H. G. Brooke, of Manchester, read a valuable and instructive paper at the International Medical Congress at Berlin in 1890, in which he set himself to consider the *ætiology* of drug eruptions. His special purpose was to deal with the question, "Are the dynamic eruptions of Behrend to be separated completely from those which he considers specific?"

Brooke first refers to the fact that so many substances of "totally dissimilar chemical constitution" are able to produce one and the same exanthem. And, again, that "the same substances produce in different individuals very different eruptions."

He points out the never-to-be-forgotten influence of idiosyncrasy as it exists in the individual, and discusses the suggestion of Behrend that "drugs do not produce their cutaneous phenomena by direct action, but through the agency of a limited number of chemical substances (now called toxins) which are engendered in the body under their influence." By this hypothesis, says Brooke, "the number of the causes of drug eruptions are reduced, and, according to Behrend, the question becomes chiefly one of pathological anatomy."

He then proceeds to describe those drug exanthems which are chiefly angioneurotic in character, apart from those which are of inflammatory kind, and have the quality of new growths.

### "Selective Action."

In relation to those which are chiefly of nervous origin he admits the possibility of specific action (in the sense that Behrend uses the term), if we study the influence of drugs upon the different functions of the nerves which supply the heart—digitalis for example—and recognises the uniform "selective action" they have on special nerves supplying it. In illustration of those drugs, the action of which is less uniform and regular, he mentions nicotine, which, as we are aware, "varies so much in its effect in different people." Again, in a third group, he refers to drugs which are "so inconstant in their action that their effects cannot be anticipated."

In this idiosyncrasy is to be recognised. The larger number of the angioneurotic group, the manifestations of which are uncertain and irregular, depend upon the individual affected, quinine, chloral and antipyrin being cited as examples. Whatever the varying eruptions may be in position, form, extent, duration, and degree, they depend upon the particular structures involved, whether central or peripheral.

Brooke concludes by stating that he "does not discover any clearly-defined differences between the specific and dynamic rashes of Behrend, but rather a continuous and gradually progressive series extending from those which occur with almost absolute regularity to those which vary in every individual." Neither, *per contra*, can he believe that "the limitations in the *modus operandi* of the drugs are completely accounted for by the special influence exerted on particular parts of the cutaneous nerve system."

So far as the vascular system is concerned in the production, he considers that the "general influence of all the drugs must be confined to the production of hyperæmia."

### Skin Peculiarities.

"The cutaneous blood-vessels are imbedded in a richly organised mass of tissue of extraordinary individual variety, thus explaining the strikingly different lesions which occur among the angioneurotic exanthemata. When a simple paralysis occurs we have a simple erythema, which can, when the epidermis has little power of resistance, give rise to vesiculating and bullous erythemata; or, again, when a spastic element concurs the erythematous basis may develop into papules like those of erythema multiforme and erythema nodosum, or into wheals; or, finally, where a passive condition (Stanung) takes the place of the active hyperæmia, purpura or œdema may be produced. All these different types depend, not upon the constitution of the vascular and nervous systems, but upon the peculiarities of the skin itself, and vary according to its individuality. The remaining number of the group (as in a quinine rash when a long-standing hyperæmia of more inflammatory kind with desquamation occurs) are explained by a local direct influence of the drug circulating in the blood on the tissues of the skin themselves over and above the angioneurotic influence of the nerve centres." (Brooke.)

An important contribution was also made to the same Congress by Dr. Fox, of London, whose purpose was more particularly to consider the consequences of the *local* application of certain drugs (such as iodoform, sublimate, belladonna, chrysarobin, etc.), and he deals with the question, "Are the widespread erythematous and vesicular eruptions arising from the above-named to be placed in the same category with the universal eruptions following the internal use of drugs?" After reviewing the writings of many authors he discusses the subject under the following heads:—

1. Effects strictly local and limited to those regions with which the drug comes into contact, due directly to superficial local irritation, and occurring without any systemic reaction.
2. Effects also local, and due to peripheral action, but not coterminous with the site of application of the drug, and radiating for some distance around; occurring with or without systemic reaction.
3. Radiating effects due to more central irritation, with or without coincident systemic reaction.

In the first group is included the substances which produce a local dermatitis (the dermatitis venenata of White, of Boston, who has written exhaustively on this subject).

Special mention is made of mercurial preparations, belladonna, chrysarobin, arnica, resorcin, and iodoform, besides other agents, as lime, sugar, etc., when there is no absorption.

General erythematous eruptions sometimes followed local applications of belladonna, and mercurial inunctions were occasionally accompanied by a general exfoliative dermatitis.

### An Instructive Case.

Here I will mention an instructive case which occurred to me some years ago. A gentleman who had been under medical care for about a week with a limited urethritis of gonorrhœal origin had diligently used a solution of permanganate of potash, both as a wash to the glans penis and also for urethral injection. Rather earlier than the time when a primary chancre would have developed had he contracted syphilis, he exhibited a raised indurated sore upon the preputial mucous membrane, of such questionable character that it was impossible from appearance alone to decide its nature. The use of the solution was continued, and a few days later he presented himself with two more sores, of equally uncertain nature, at the base of the glans. Their rapid development and unusually inflammatory character induced me to question him closely, especially as he admitted that he suffered pain on several occasions after the use of the lotion. It then transpired that he had prepared his own solution, and had used it with a small sponge before the crystals had dissolved, with the result that one or two adhered to the surface and had produced the sores complained of. Since then I have met with cases in which women had, with a Higginson's syringe, injected undissolved crystals of the same kind into the vagina for the treatment of leucorrhœa, and had produced inflammatory irritation.

In the second group Fox describes the effects of iodoform, and Campbell Pope's experience (as reported in the *British Medical Journal*, April 6, 1889) is referred to, in which an "eczema-like" dermatitis attacked the whole limb on three different occasions when iodoform gauze or ointment, separately used had been applied for the treatment of an inflamed bursa of the arm.

### Further Examples.

Taylor, of New York, has recorded a case in which the ethereal solution of iodoform applied to chancroids on the prepuce produced rapid swelling and inflammation of the whole penis. Marcus Beck had a similar experience after operation for removal of scirrhus mammæ. Hebra, Tilbury Fox, Durhing, Cartier and others have reported similarly. In 1897 Wathen, of Clifton, published an interesting "Personal Reminiscence of the Local Effects of Iodoform." On one occasion the appearance and course of the eruption are said to have suggested hydroa herpetiforme, and on another some of the characteristics of cheiro-pompholyx developed. Numerous instances of the kind have since been published by other observers, and I have had under my own care several cases of similar nature.

An interesting case is published in the *Practitioner* (1886). A wound on the skin of a girl was freely dressed with iodoform, and subsequently the entire forearm inflamed, and much febrile reaction followed in spite of the discontinuance of the drug. The rash spread to the face, neck, and chest, consequent upon absorption.

The forementioned are examples of Fox's first subdivision of the third large group constituting "the eruptions which begin locally from peripheral irritation and spread, and are followed by the evolution of remotely situated areas which may join together with the originally inflamed area to form a more or less generalised eruption."

His second subdivision, viz. "more or less widely-spread or generalised eruptions, beginning either near the site of application of the drug, or remotely in several places without local signs," is illustrated by the experiences of Zeissl, C. J. Taylor, Berkeley Hill, Bolowski, and others.

(*To be continued.*)

---

### DISEASES OF THE SKIN.

(*A Method of Investigation suggested by* Dr. Stowers *to assist the Student.*)

Sex—Age—Name of Patient—Address—Single or Married—Number of Children, Miscarriages, &c.—Nature of Occupation—Whether Vaccinated and Re-Vaccinated—Mode of living, as to Food, Alcohol, Exposure, &c.—(*if infant, whether breast or hand-fed*)—Present condition of General Health, Nutrition, &c.

Previous History—(Rheumatism, Gout, Syphilis, or severe illness)—Past Residence, as to Climate, &c.

Family History—Others affected—Evidence of Infection or Contagion.

Duration of Disease—Single or multiple, limited or universal—Predilection for certain sites of body—Mode of onset—Continuous or Intermittent—Mode and rate of progress.

Objective Symptoms (*N.B.—Examine the whole body and scalp*)—Primary or Initial Lesions (description of)—Uniformity, symmetry, or asymmetry—Polymorphism.

| *Essentials.* | *Accidentals.* |
|---|---|
| Macule or stain. | Resulting from congestion or inflammation in relation to friction, scratching, soaps, irritants (simple or chemical), caustics, clothes, retained secretions, traumatism, &c. |
| Papule. | Scale. |
| Vesicle. | Crust. |
| Bleb. | Fissure. |
| Pustule. | Excoriation. |
| Wheal. | Ulcer. |
| Tubercle. | Scar. |
| Tumour. | |

Subjective Symptoms — Pain — Tingling — Itching—Diminished sensation (anæsthesia)—Augmented sensation (hyperæsthesia).

Constitutional Symptoms or Disturbances—Pulse—Respirations—Temperature.

Bowels, action of—(*Constipation—Diarrhœa—Character of Fæces* (*Worms?*), &c.).

Urine, characters of (*detailed Examination necessary, including Specific Gravity*).

(Exanthemata?)

(Malingering?)

(Examination of Clothes?)

Previous Treatment (effects of remedies)—*a.* General—[Drug Eruptions? — (*Dermatitis Medicamentosa*)]. *b.* Local—[Effects of irritants?—(*Dermatitis Venenata*)].

Microscopic Characters.

Complications.

Diagnosis.

Treatment required (Palliative and Curative)—*a.* General. *b.* Local.

Prognosis.

Result.

